# Predicting domain-domain interactions using a parsimony approach

**DOI:** 10.1186/gb-2006-7-11-r104

**Published:** 2006-11-09

**Authors:** Katia S Guimarães, Raja Jothi, Elena Zotenko, Teresa M Przytycka

**Affiliations:** 1 National Center for Biotechnology Information, National Library of Medicine, National Institutes of Health, Bethesda, MD 20894, USA; 2 Center of Informatics, Federal University of Pernambuco, Recife, PE 50732, Brazil; 3 Department of Computer Science, University of Maryland, College Park, MD 20742, USA

## Abstract

We propose a novel approach to predict domain-domain interactions from a protein-protein interaction network. In our method we apply a parsimony-driven explanation of the network, where the domain interactions are inferred using linear programming optimization, and false positives in the protein network are handled by a probabilistic construction. This method outperforms previous approaches by a considerable margin. The results indicate that the parsimony principle provides a correct approach for detecting domain-domain contacts.

## Abstract 2

A new parsimony approach for the prediction of domain-domain interactions is presented and demonstrated to provide improvement in prediction coverage and accuracy.

## Background

Knowledge about protein interactions helps provide deeper insights into the functioning of cells. Protein interaction data are collected from various studies on individual biological systems, and, more recently, through high-throughput experiments, such as yeast two-hybrid and tandem affinity purification followed by mass spectrometry [1-8]. This rapidly growing collection of protein-protein interaction data provides a rich, but quite noisy, source of information [9-12], and is being analyzed with increasingly sophisticated computational methods.

Proteins typically contain two or more domains. About two-thirds of proteins in prokaryotes and four-fifths in eukaryotes are multidomain proteins [13]. Interaction between two proteins typically involves binding between specific domains, and identifying interacting domain pairs is an important step towards understanding protein interactions and the evolution of protein-protein interaction networks. Many groups have contributed computational methods aimed at discovering interacting domain pairs [14-23]. With the exception of [23], they all rely on protein-protein interaction networks.

Many domain-domain interaction prediction methods tie the goal of predicting domain interactions to the seemingly related goal of predicting protein-protein interactions. For example, the Association method [15] scores each domain pair by the ratio of the number of occurrences of a given pair in interacting proteins to the number of independent occurrences of those domains. This score can be interpreted as the probability of interaction between the two domains. Several related methods have also been proposed [18,19]. Deng and colleagues [16] extended this idea further and applied a maximum likelihood estimation approach to define the probability of domain-domain interactions. Their expectation maximization algorithm (EM) computes domain interaction probabilities that maximize the expectation of observing a given protein-protein interaction network. Other groups proposed alternative methods for this task: linear programming [20], support vector machines [14], and probabilistic network modeling [17].

Nye and colleagues [21] evaluated the correctness of those domain-domain interactions predicted by the Association method, the EM method, and their own lowest p value method. For this, they used interacting protein pairs with crystal structure evidence to test the correctness of the predicted domain interactions. They divided the test set of interacting pairs of proteins into groups depending on the number of potential candidate domain pairs. Interestingly, for the largest group of protein pairs all methods were outperformed by a Random method, exposing their shortcomings.

More recently, Riley and colleagues [22] introduced a new method, called the Domain Pair Exclusion Analysis (DPEA), to predict domain-domain interactions. DPEA is based on computing an E-value, which measures the extent of the reduction in the likelihood of the protein-protein interactions network, caused by disallowing a given domain-domain interaction. This is assessed by comparing the results of executing an expectation maximization protocol under the assumption that all but the given pair of domains can interact. DPEA outperforms the Association and EM methods by a significant margin in the number of recovered domain-domain interactions confirmed by Protein Databank (PDB) [24] crystal structures.

In this work, we explore an alternative model for predicting domain-domain interactions. In our approach, we completely decouple domain-domain interaction prediction from protein-protein interaction prediction. We hypothesize that interactions between proteins evolved in a parsimonious way and that the set of correct domain-domain interactions is well approximated by the minimal set of domain interactions necessary to justify a given protein-protein interaction network. We refer to our approach as the 'Parsimonious Explanation' (PE) method. We formulate PE as a linear programming optimization problem, where each potential domain-domain contact is a variable that can receive a value (called the 'linear program (LP)-score'), ranging between 0 and 1, and each edge of the protein-protein interaction network corresponds to one linear constraint. This formulation allows for a novel way of handling the noise (false positives) in the protein interaction data. Namely, we construct a set of linear programming instances in a probabilistic fashion, in which the probability of including an LP constraint equals the probability with which the corresponding protein-protein interaction is assumed to be correct, and average the results to get the LP-score for each pair.

To control for possible over-prediction of interactions between frequently occurring domain pairs, we assign a promiscuity versus witnesses (pw)-score to every predicted domain-domain interaction. The pw-score, derived from two observations, measures the confidence in the prediction. First, domain-domain interactions that have many witnesses (interacting pairs of single domain proteins that support it) are more likely to be correct than ones that have a few or no witnesses. Second, there are promiscuous domain-domain interactions that are scored high due to the frequency of their appearance and not to the specific topology of the protein-protein interaction network. In view of these observations, the pw-score formulation rewards domain interactions that have many witnesses and penalizes promiscuous interactions.

We assess the performance of our method with two different types of evaluations. Our first evaluation, which is very similar to that done by Riley and colleagues [22], documents the fraction of predictions confirmed to interact (based on PDB [24] crystal structures, as inferred in iPfam [25]). We compare the performance of the PE and previous methods by plotting curves of prediction accuracy versus their coverage. This type of evaluation shows that PE outperforms other methods. We also compare PE directly with DPEA, shown to be the best among the currently available methods, using the number of confirmed interactions among the 3,000 top-scoring predictions, separating them into easy and difficult predictions. In the easy category are domain pairs for which there is at least one witness. Interacting domain pairs that do not have such direct experimental evidence fall under the difficult category, as they are hard to detect for any method. The PE method recovers more experimentally confirmed interactions in both classes. In particular, in the difficult class, it outperforms DPEA by an order of magnitude.

Our second type of evaluation of the PE method involves finding whether or not the predicted domain pairs do, in fact, mediate interactions between specific protein pairs. In other words, given a protein-protein interaction, we are interested in finding whether the highest scoring domain pair between those proteins is, in fact, known to interact. If it does, then we consider our prediction to be correct. In case of multiple highest scoring pairs, each one of them is considered in the evaluation. This type of 'protein interaction specificity' evaluation has been used before [21]. For this evaluation, we used only those protein-protein interactions containing multiple domain pairs, at least one of which is in the gold standard set. A pair of proteins, P and Q, is said to contain domain pair (x, y) if domain x is present in protein P and domain y is present in protein Q, or vice versa. In this experiment, the PE method reached estimated values of 75.3% for positive predictive value (PPV) and 76.9% for sensitivity, while DPEA presented an estimated PPV of 42.5% and sensitivity of 36.9%.

## Results and discussion

We applied the PE method on a protein-protein interaction dataset comprising 26,032 interactions underlying 11,403 proteins from 69 organisms. This set was constructed by Riley and colleagues [22] from the Database of Interacting Proteins (DIP) database [26]. Protein domains were annotated using Pfam hidden Markov model (HMM) profiles [27].

The PE method assigns a LP-score and a pw-score to each potential domain-domain interaction. Intuitively, the LP-score estimates the potential of a given domain pair in explaining protein interactions, based on the overall goal of parsimony principle, while the pw-score factors in the influence of the number of occurrences of a pair in the data set, and the number of witnesses present. Potential interactions whose LP-scores are above a certain threshold and whose pw-scores are below another threshold are predicted to be putative interactions. We model the experimental error (false positives) in the protein-protein interaction network by a probabilistic construction of the linear program, as described in Materials and methods.

**Figure 1:**
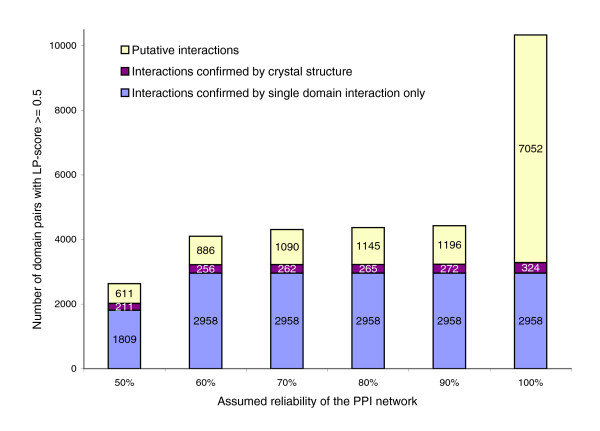
Influence of assumed network reliability on LP-score predictions. Influence of the assumed network reliability on the number of pairs with LP-score above 0.5 and the number of interactions among those that are confirmed by crystal structures in our gold standard set or by witnesses. The number of pairs confirmed by the gold standard set remains stable for all network reliability assumptions, and interactions predicted under assumption of a lower network reliability almost always are a subset of the interactions predicted under the assumption of a higher network reliability.

We performed experiments with assumed reliabilities of 50%, 60%, 70%, 80%, 90%, and 100%. The most tangible general effect of increasing the assumed network reliability is an increase in the LP-scores, resulting in a higher coverage, but with lower prediction accuracy with respect to the set of interactions confirmed by crystal structures. Figure 1 shows the influence of the assumed network reliability on the number of pairs with LP-score above 0.5 and the number of interactions confirmed by crystal structures in our gold standard set or by witnesses. The number of such pairs confirmed by crystal structures remains stable for all network reliability assumptions. Furthermore, the set of high scoring (LP-score close to 1) interactions remains stable. That is, interactions predicted under assumption of lower network reliability almost always are a subset of the interactions predicted under the assumption of a higher network reliability. This demonstrates the robustness of the PE method with respect to the reliability of the underlying protein-protein interaction network.

**Figure 2:**
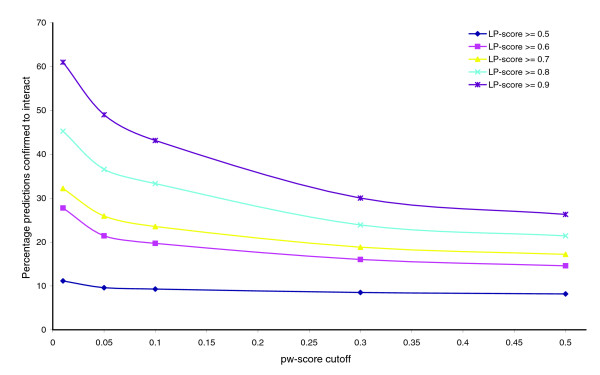
Influence of pw-score cutoff on accuracy of predictions. A pw-score close to 1 indicates a promiscuous domain pair that can obtain a high LP-score independent of the topology of the underlying protein-protein interaction network, and does not have significant witness support. Higher LP-score cutoffs lead to higher prediction accuracy; smaller (more stringent) pw-score cutoffs help improve it further.

The pw-score is an indicator of the possible over-prediction of interactions between domains that occur frequently, which also takes into account the number of witnesses for that given pair in view of the assumed reliability of the network. More precisely, for a given domain pair, the pw-score is the minimum of a p value (which measures the probability of obtaining the same or higher score in a random network of interactions for the same protein set) and a probability based on witness support and the network reliability rate (see Materials and methods). A high LP-score can be due to the sheer number of occurrences of the given domain pair in proteins included in the interaction network. However, we verified that many promiscuous domains do interact despite of a high p value. To detect such interactions, we rely on the evidence from the set of witnesses. The confidence in the witness is a function of network reliability as described in Materials and methods. The role of the pw-score is to allow some control over these factors. A pw-score close to one indicates a promiscuous domain pair that can obtain a high LP-score independent of the topology of the underlying protein-protein interaction network, and does not have significant witness support. Choosing a smaller (more stringent) pw-score cutoff naturally leads to higher prediction accuracy, as can be seen in Figure 2.

Based on observations that the reliability of high-throughput protein-protein interaction networks is about 50% [9-11], we have chosen to report the results based on 50% network reliability. Our predictions are filtered to exclude those that have a pw-score greater than a chosen cutoff. Those predictions that have higher pw-scores are considered to be statistically insignificant. We analyzed our results for pw-score cutoffs of 0.01 and 0.05. These cutoffs were chosen to demonstrate the ability of the PE method to recover difficult domain pairs confirmed to interact. A higher pw-score cutoff would lead to many more domain pairs being predicted among those with high LP-scores due to the possibility of them being confirmed by a number of witnesses. Since truly interacting pairs may or may not be promiscuous, and may or may not have witnesses, the choice of the appropriate pw-score cutoff should, if possible, be made with this issue in mind with regard to the family of particular interest. We report as supplementary material the 3,000 highest scoring (LP-score) domain pairs with pw-score cutoffs of 0.01 (Additional data file 1) and 0.05 (Additional data file 2) from our experiments with a network reliability of 50%, which were used for our analysis. We also provide two sets of predictions from LP-score experiments with network reliabilities of 50% (Additional data file 3) and 60% (Additional data file 4); the first contains 3,610 domain pairs, and the latter has 3,944.

### Enrichment of confirmed interactions in high-scoring domain pairs

Motivated by Riley and colleagues [22], we developed experiments to evaluate the performance of our method based on the number of high-scoring domain-domain interactions confirmed by the gold standard set, which is a set of pairs confirmed to interact, as inferred in iPfam [25] based on PDB crystal structures. This set is described in Materials and methods, and a list of the 783 pairs occurring in our dataset is available as Additional data file 5.

**Figure 3:**
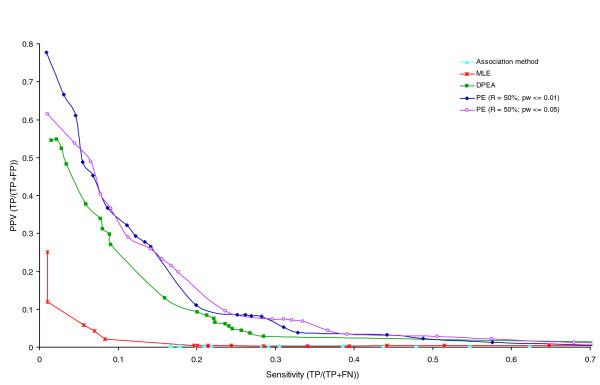
PPV versus sensitivity in enrichment of confirmed interactions experiment. Comparison of PPV (TP/(TP + FP)) and Sensitivity (TP/(TP + FN)) attained by the PE method with pw-score cutoffs of 0.01 and 0.05, and previously by the Association, EM, and DPEA methods. The comparison is based on estimations of how many of the high-scoring domain-domain interactions are confirmed by the gold standard set.

We compared the PE method with previous methods (Association, EM, and DPEA), by plotting curves of their positive predictive value versus their sensitivity. The comparison plot is given as Figure 3; the details on the estimation can be found in Materials and methods. Due to the relatively small number of interactions confirmed by crystal structures, the rate of false positives may be excessive. Although the estimated measures may be impaired by this, they still show that PE clearly outperforms other methods by a considerable margin.

We also performed a comparison of the number of predictions by the PE and the DPEA methods confirmed to interact based on crystal structure evidence; we analyzed easy and difficult predictions separately. The necessity of evaluating predictions based on how difficult they are to predict has been justified before [22]. To separate the easy predictions from the difficult ones, Riley and colleagues [22] associate with each domain a measure called 'modularity', which is equal to the average number of domains in proteins containing the given domain. A non-trivial prediction would then involve at least one domain, out of the pair, with modularity of at least 2.0. This, however, does not exclude the possibility that a given domain pair has a witness that would make the prediction significantly easier; additionally, even an isolated occurrence of a domain in a protein with a large number of domains increases the modularity of the domain significantly, without necessarily making the prediction process more difficult. Therefore, we adopted a much more stringent classification of easy and difficult predictions. A domain-domain interaction is considered to be difficult to predict (from the underlying protein-protein interaction network) if there is no interacting pair of single domain proteins containing respective domains.

**Figure 4:**
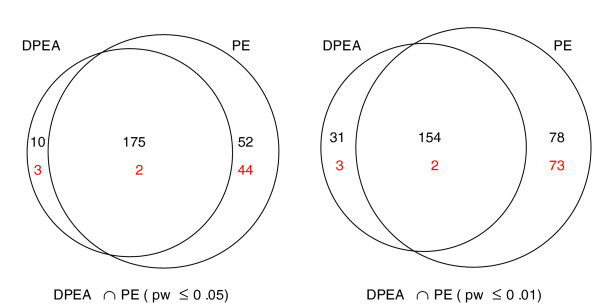
Comparison of gold standard pairs recovered by PE and DPEA. Comparison between the sets of gold standard pairs recovered among the 3,005 pairs considered as high-confidence predictions of the DPEA method and among the 3,000 top scoring pairs selected by the PE method with pw-score cutoffs of 0.01 and 0.05. In red are the numbers of difficult gold standard pairs predicted. In the set of 185 gold standard interactions recovered among the 3,005 high-confidence domain pairs by the DPEA method, only 5 are in the difficult category. In comparison, among the 3,000 top scoring domain interactions reported by the PE method with a pw-score cutoff of 0.05, there are 46 difficult pairs (75 difficult pairs with cutoff 0.01).

Figure 4 shows the comparison of the sets of gold standard pairs recovered among the 3,005 pairs considered as high-confidence predictions by the DPEA method and those among the 3,000 top-scoring pairs selected by the PE method with pw-score cutoffs of 0.01 and 0.05. We indicate the number of difficult gold standard pairs predicted in red. We note that, out of 185 gold standard interactions recovered among the 3,005 high confidence domain pairs by the DPEA method, only 5 are in the difficult category. In comparison, among the 3,000 top-scoring domain interactions reported by the PE method with a pw-score cutoff of 0.05, there are 46 difficult pairs (75 difficult pairs with a pw-score cutoff of 0.01).

### High scoring putative interactions

In Table 1, we list the 50 highest-scoring (LP-score) predictions with a pw-score ≤ 0.01. Among these predictions, only 17 are not in the gold standard set and 14 pairs that are in the difficult category. Nine of these difficult predictions are confirmed by crystal structures and three have been inferred to interact in the literature [28-30]. The last one, involving cyclin and cyclin-dependent kinase regulatory subunit (CKS), has been investigated by Aloy and Russell [31]. They proposed that the CKS/cyclin interaction may be indirect and may involve CDK2 as an intermediate protein, contrary to the information in the high throughput interaction data. Therefore, if Alloy and Russell's hypothesis is correct, then our prediction will turn out to be wrong.

### Predicted interaction partners for the Ras and SNARE families of domains

In Table 2, we provide a list of interaction partners for the Ras and SNARE domain families. The Ras domain belongs to a large super-family of G-proteins, which bind guanine nucleotides (GTP and GDP). Ras acts as a switch, which in its resting state is in a complex with GDP, and in its active state is bound to GTP. The activity of the Ras switch is controlled upstream by proteins called exchange factors by nucleotide exchange reaction between GDP and GTP. The signal is subsequently passed downstream of the signaling cascade. Ras regulates many aspects of cell growth and differentiation, cytoskeletal integrity, proliferation, cell adhesion, apoptosis, and cell migration. Ras and Ras-related proteins are often deregulated in cancers, leading to increased invasion and metastasis, and decreased apoptosis. Thus, understanding interactions between the Ras homology domain and other proteins is of primary interest. Out of 35 Ras putative interactions with a LP-score ≥ 0.5 and a pw-score ≤ 0.05, six are difficult and three (among them one difficult) are documented by crystal structures. More than 70% of the easy predictions belong to the high-confidence DPEA predictions. (We note that the PE predictions with a LP-score below 0.6 are also border-line predictions for DPEA.) The interaction between Ras and Mss4 is known from the literature, with the caveat discussed below.

The SNARE domain (Pfam PF05739) is thought to act as a protein-protein interaction module in the assembly of a SNARE protein complex. Out of the 223 potential domain pairs in our dataset involving SNARE, almost all of which are difficult, only 5 are in the gold standard set. All but one of the PE method's eight predictions of SNARE interactions are in the difficult category, and four of them are documented by crystal structures.

When interpreting the results for such families, one has to keep in mind that the PE method predicts domain interactions based on the evidence found in the underlying protein interaction dataset, that is, a predicted domain interaction is expected to mediate at least one protein-protein interaction in the dataset. Large superfamilies like Ras contain several related but yet different subfamilies, such as Ras, Rab, Rac, Ral, Ran, and so on. Since Pfam has classified all Ras-type families into one big superfamily based on their sequence similarity, a prediction between Ras and Mss4 does not necessarily mean that all subfamilies interact with Mss4; it only means that there is at least one subfamily in the Ras superfamily that is predicted to interact with Mss4. Since Ras and SNARE are large domain families, to recover true interactions, many of which may have high pw-scores, we used a pw-score cutoff of 0.05 to construct Table 2. One needs to keep in mind that predicting interaction for promiscuous domains could be difficult for the PE method, as a lower pw-score cutoff may not recover all true interactions while a higher pw-score cutoff may lead to spurious predictions, reducing the prediction accuracy.

### Predicting interacting domain pair(s) within a given interacting protein pair

Given a pair of interacting proteins, predicting the domain pair(s) that mediate the interaction is a problem that has been studied before[21]. In order to assess and compare the performance of the PE and other domain interaction prediction methods for this particular problem, we assumed that, if an interacting protein pair contains domain pairs that are confirmed to interact (by crystal structure evidence), then this protein-protein interaction is mediated by (possibly more than one) such confirmed domain-domain interactions. Therefore, for this experiment, we restricted our attention to only those interacting protein pairs that contain at least one gold standard domain pair that could mediate the interaction, and tested whether this pair(s) received the highest score among all domain pairs that can potentially mediate a given protein interaction. In Material and methods we discuss further the protein pairs selected for this experiment. The set of 1,780 interacting protein pairs used for this experiment is available as Additional data file 6.

We estimated the PPV and the sensitivity of the Association, EM, PE, and DPEA methods, and we also estimated the performance measures that could be expected by chance using a Random method (for details, see Materials and methods). The results for PE with pw-score cutoffs of 0.01 and 0.05 were very close, so we present only one set of numbers. The scores for the Association, EM, and the DPEA methods were taken from those generated by Riley and colleagues [22].

**Figure 5:**
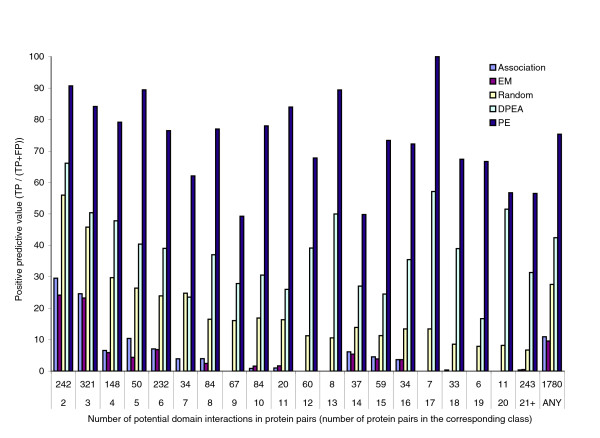
Comparison of positive predictive values in mediating domain pair prediction experiment. Estimated positive predictive value of the Association, EM, PE, and DPEA methods, and the performance expected by chance in such experiments, called the Random method. The results are presented according to the number of potential domain-domain interactions between the protein pairs in the set, and also in general. The numbers along the x-axis represent the number of protein pairs in the corresponding class. The PE method outperforms the previous methods in every class. In particular, for the 242 protein pairs with only 2 potential domain-domain interactions, PE has a PPV of 90.7%, and sensitivity of 93.8%, and for the 993 protein pairs with 2 to 6 potential domain-domain interactions, the PE method consistently has an average PPV above 76%. Overall, the PE method has an estimated average PPV of 75.3%. The Association and the EM methods both perform worse than Random; possible reasons for such an outcome are discussed in the text.

In Figure 5, we present the PPV values, according to the number of potential domain-domain interactions between the protein pairs in the set, similar to those in Nye and colleagues [21], and also in general. The numbers on the x-axis indicate the quantity of protein pairs in the corresponding subgroup. The PE method outperforms all the previous methods in every class, both in terms of prediction accuracy as well as the coverage. In particular, for the set of 242 protein pairs with only 2 potential domain-domain contacts, PE has a PPV of about 91% and a sensitivity of about 94%, and for the set of 993 protein pairs with 2 to 6 potential domain-domain contacts, the PE method has a PPV and a sensitivity of at least 76%. For the set of 243 protein pairs with more than 20 potential domain-domain contacts, PE has a PPV and a sensitivity of at least 56.5%. Overall, based on this measure, the PE method has an estimated average PPV of 75.3%, against 42.5% for the DPEA method, while the estimated sensitivity for the PE method was 76.9%, more than twice that for the DPEA method (36.9%).

We observed that the Random method outperforms both the Association and the EM methods. This is not surprising considering the fact that it has been shown before[21] that Random performs as well as these two methods. However, we found it interesting that the Association method actually outperforms the EM method, which contrasts Nye and colleagues' [21] observations. The reason for the dominance of the Association method over the EM method could be attributed to the latter's preference for domain pairs involving Pfam-B domains. Since our gold standard set of positives only contain Pfam-A domains, many of the EM method's high-scoring predictions containing Pfam-B domains are classified as false-positives.

Below we present some additional discussion on the performances observed. A plot similar to Figure 5, depicting the results of the estimated sensitivity measures in this experiment, is available as Additional data file 7.

### Rationale behind the performance of the PE method

**Figure 6:**
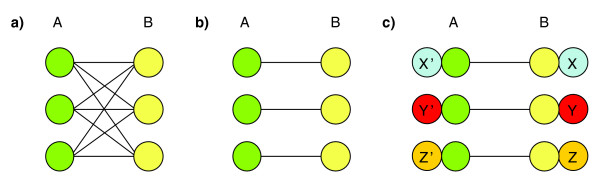
Specificity of interactions. **(a) **A hypothetical subnetwork for non-specific interaction between proteins containing two domains: each protein containing domain A interacts with each protein containing domain B. Detecting such interactions is easy for all four methods: Association, EM, DPEA, and PE. **(b) **A hypothetical subnetwork for highly specific interactions between proteins containing domain A and proteins containing domain B. Since only a small number of interactions actually occur, out of all possible interactions between pairs of proteins containing domain pair {A, B}, detecting such specific interactions is difficult for the EM and the Association methods, but not for the DPEA and the PE methods. **(c) **Hypothetical subnetwork for highly specific interactions in the context of multidomain proteins. PE will attribute these interactions to domain pair {A, B}, as it requires prediction of one interaction {A, B} to justify three protein-protein interactions. On the other hand, the association and the EM method will assign higher probability to domain pairs {X, X'}, {Y, Y'}, and {Z, Z'}, as it is beneficial to assign higher probabilities to interactions involving rare domains, that is, X, Y, and Z.

There are two main reasons for the PE method's improved performance, both of which relate to interaction specificity. An ideal example of a non-specific interaction between domains A and B is illustrated in Figure 6a. A non-specific interaction corresponds to a complete bipartite graph where the proteins containing domain A comprise one set of the bipartition, and the proteins containing domain B comprise the second set. If the interaction is fully non-specific, then all proteins with domain A would interact with all proteins with domain B. The more specific the interaction, the sparser is the interaction graph. In the case of a highly specific interaction there is a one-to-one correspondence between interacting proteins, as illustrated in Figure 6b. Since the EM method considers each missing edge as evidence that the interaction did not occur, for every specific interaction, the support for the observation that the two domains do not interact is much higher than the support for the observation that they do interact. This problem is carefully avoided in the DPEA method with the help of the E-value measure. In the PE method this is never a problem, as it does not consider lack of interaction as support for non-interaction.

**Figure 7:**
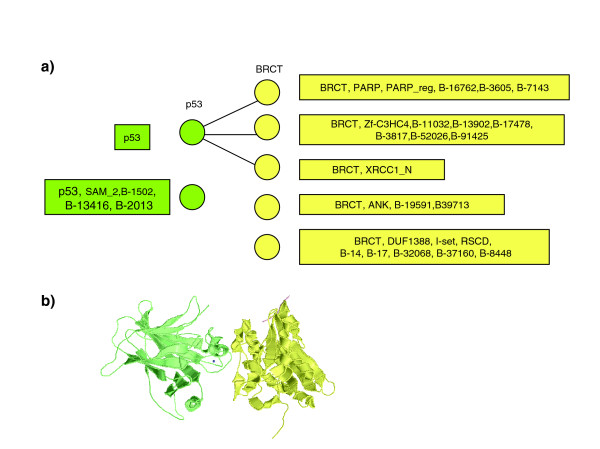
P53-BRCT interactions. **(a) **The subnetwork of the protein-protein interaction network spanning only the human proteins with p53 and BCRT domains. Three pairs of these proteins interact (as indicated by connecting edges). The domain composition of each protein is given in the corresponding box. PE correctly identifies BRCT-p53 as interacting partners. **(b) **Crystal structure of the p53-BCRT complex (PDB entry 1gzh); only the p53 and BRCT domains are shown in the figure.

The second shortcoming with machine learning methods, which are trained best to predict the protein interaction network, is their tendency to use infrequent domains to justify interaction between multi-domain proteins. Consider a hypothetical situation where a set of proteins containing domain A interacts with a set of multi-domain proteins containing domain B (Figure 6c). If domains accompanying domain B in multi-domain proteins are infrequent, then it is beneficial from the perspective of the expectation maximization to assign higher interaction probability to the pairs involving rare domains, that is {*X*,*X*'}, {*Y*,*Y*'} and {*Z*,*Z*'}, respectively. We call this effect 'a shift towards rare domains' phenomenon. Since the PE method seeks an explanation that involves the smallest possible (weighted) number of domain pairs, it is immune to the shift towards the rare domains phenomenon. Figure 7 illustrates this situation on a real example involving p53 and BRCT domains. Domain p53, also known as tumor protein 53 (TP53), is a transcription factor that regulates the cell cycle, and hence functions as a tumor suppressor. It is very important for cells in multi-cellular organisms to suppress cancer. The BRCT domain is important for its function in DNA repair and transcriptional activation. The interaction between these two domains has been documented by a crystal structure in the PDB (PDB ID 1gzh). Since BRCT is involved in other interactions not involving p53, the BRCT-p53 interaction remains undetected by the EM method. This interaction also remains undetected by the DPEA method, most likely because it has no witnesses, and in the absence of one or more witnesses DPEA seems to be affected by a shift towards the rare domains phenomenon. However, the PE method recovers this domain-domain interaction with a LP-score of 0.627 and a pw-score equal to zero.

Based on the mathematical formulation of the PE method, one may be concerned about possible over-prediction of interactions between frequently occurring domains. To address this question, we introduced the pw-score as a measure of confidence in our prediction. With the assumed network reliability of 50%, about 10% of the gold standard pairs achieve a pw-score >0.05, and about 25% of the gold standard pairs achieve a pw-score >0.01, hence those pairs cannot be recovered. Since the number of the promiscuous domain pairs is relatively small, false-positives between them are easier to detect, and subsequent knowledge on 'non-interaction' between such domains can be included in the model.

## Conclusion

We present a new method for identifying interacting domain pairs. The method, abbreviated as PE, is based on the parsimony principle: domain-domain interaction partners are predicted by identifying the minimal weighted set of domain pairs that can justify a given protein-protein interaction network. The corresponding optimization problem is formulated using linear programming. Our results show that the PE method outperforms previous methods considerably. The most dramatic improvement is evident in the recovery of known true domain-domain interactions that are considered to be difficult to recover.

We estimate PPV and sensitivity of our method to be 75.3% and 76.9%, respectively. However, one has to keep in mind that such estimations are, in this case, very difficult due to lack of interaction data. Our test set for this experiment makes the assumption that domain-domain interactions that have been proven to mediate a specific protein-protein interaction are also likely to mediate other protein interactions that contain those domain pairs. In this case it is reasonable to presume that domain pairs not in the gold standard set do not interact in the context of the given protein pairs. Nonetheless, there may be cases where that is not true; therefore, the reported numbers should be considered as estimates.

Our method provides a unique way to represent uncertainties of the protein-protein interactions in a high throughput protein-protein interaction network. In this work, we assumed that the probability of error for each protein-protein interaction represented in the network is the same. However, our approach can also be applied when probability of correctness of each interaction is assessed individually, based on the type of experiment used for its detection and other supplementary information. For example, the confidence values, based on logistic regression, assigned to links in the network by Bader and colleagues [12].

The PE method is a significant departure from the underlying assumption of the EM method. While EM methods work well for the problem of identifying interacting protein pairs based on their domain composition, it does not provide an effective approach to detecting interaction between domains [21,22]. We showed that the PE method performs significantly better than the DPEA method, which has been demonstrated to be better than other previous methods. These results provide an argument behind the correctness of the parsimony principle in detecting domain-domain interactions based on the topology of the protein-protein interaction network.

## Materials and methods

### Data set and gold standard set selection criteria

We used the protein-protein interactions and the protein domain composition dataset used by Riley and colleagues [22]. This set was obtained from the DIP database[26], with added domain annotation from Pfam HMM profiles, and contained 26,032 interactions underlying 11,403 proteins from 69 organisms. The domain-domain interaction pairs confirmed by PDB crystal structures were obtained from the iPFAM database [25] (December 2005 version), which contained 3,074 unique domain-domain interactions. Out of those pairs, we selected as our gold standard positives interactions the 2,612 domain pairs that appear in a pair of different interacting proteins or in different chains of the same protein. Out of these, there are 783 unique domain-domain pairs actually occurring in the data set used. The list of gold standard domain-domain pairs is available as Additional data file 5.

### Evaluation experiments

We validated our method using two types of experiments. In the first experiment, we evaluated the retrieval of the gold standard positives among the top-scoring domain pairs. We used the Association, EM, and DPEA scores provided by Riley and colleagues to compare the methods by estimating their PPV:

PPV = (TP/(TP + FP))

and their sensitivity:

Sensitivity = (TP/(TP + FN))

where the number of true positives (TP), false positives (FP), and false negatives (FN) were estimated with respect to the gold standard set. One should keep in mind that, in this experiment, the set of negatives includes all potential domain pairs occurring in the dataset that are not in the gold standard set and, thus, it is most likely to contain interacting domains that have not yet been documented by crystal structures.

In the second experiment, we focused on whether the methods correctly identify domain interaction(s) mediating a given protein-protein interaction. For this part of the experiment, we selected only the set of interacting protein pairs that had at least one of the gold standard domain pairs among their potential domain contacts. To avoid distortions imposed by protein pairs with exactly one potential domain contact, only protein pairs with at least two potential contacts were considered. The set of 1,780 protein pairs used for this experiment is available as Additional data file 6; it contains a total of 2,641 occurrences of gold standard pairs.

We considered as gold standard negatives all potential domain-domain interactions that are in some protein-protein interaction of that selected set of protein pairs and do not meet the gold standard positive criteria. It is important to keep in mind that, while the gold standard set that we used is widely accepted, selection of gold standard negatives is difficult as there is no proof of non-interaction of domains.

### Linear programming formulation

Informally, we consider the problem of predicting interacting domain pairs as an optimization problem, in which the objective is to minimize the number of domain-domain interactions necessary to justify the underlying protein-protein interaction network. We formulate this problem using linear programming, in which a pair of domains *i *and *j *has a variable *x*_*ij *_if and only if the interaction data contains an interacting protein pair *P*_*n *_and *P*_*m *_containing domains *i *and *j*, respectively. Variable *x*_*ij *_represents the score of the potential interaction between domains *i *and *j*. The goal is to minimize the objective function ∑_*ij *_*x*_*ij *_subject to the set of constraints, which require that ∑i∈Pnj∈Pmxij≥1
 MathType@MTEF@5@5@+=feaafiart1ev1aaatCvAUfeBSjuyZL2yd9gzLbvyNv2Caerbhv2BYDwAHbqedmvETj2BSbqee0evGueE0jxyaibaiKI8=vI8tuQ8FMI8Gi=hEeeu0xXdbba9frFj0=OqFfea0dXdd9vqai=hGuQ8kuc9pgc9s8qqaq=dirpe0xb9q8qiLsFr0=vr0=vr0dc8meaabaqaciGacaGaaeqabaqadeqadaaakeaadaaeqaqaaiaadIhadaWgaaWcbaGaamyAaiaadQgaaeqaaOGaeyyzImRaaGymaaWcbaGaamyAaiabgIGiolaadcfadaWgaaadbaGaamOBaaqabaWccaWGQbGaeyicI4SaamiuamaaBaaameaacaWGTbaabeaaaSqab0GaeyyeIuoaaaa@4385@. Intuitively, we want to justify each protein-protein interaction, using a minimum number of domain-domain interactions possible overall.

Formally, given a protein-protein interaction network *I *= (*P*, *E*), where *P *= *P*_1_, *P*_2_,...,*P*_*N *_is the set of proteins in the network and *E *is the set of protein interactions, and a set of unordered pairs denoting all possible domain-domain interactions *D *= {{*i*, *j*}|i ∈ *P*_*n*_, *j *∈ *P*_*m*_, and *P*_*n *_and *P*_*m *_interact}, solve the linear program (LP):

Minimize ∑{i,j}∈Dxij
 MathType@MTEF@5@5@+=feaafiart1ev1aaatCvAUfeBSjuyZL2yd9gzLbvyNv2Caerbhv2BYDwAHbqedmvETj2BSbqee0evGueE0jxyaibaiKI8=vI8tuQ8FMI8Gi=hEeeu0xXdbba9frFj0=OqFfea0dXdd9vqai=hGuQ8kuc9pgc9s8qqaq=dirpe0xb9q8qiLsFr0=vr0=vr0dc8meaabaqaciGacaGaaeqabaqadeqadaaakeaadaaeqbqaaiaadIhadaWgaaWcbaGaamyAaiaadQgaaeqaaaqaaiaacUhacaWGPbGaaiilaiaadQgacaGG9bGaeyicI4Saamiraaqab0GaeyyeIuoaaaa@3F25@

Subject to: ∑(i,j)∈(Pm,Pn)xij≥1
 MathType@MTEF@5@5@+=feaafiart1ev1aaatCvAUfeBSjuyZL2yd9gzLbvyNv2Caerbhv2BYDwAHbqedmvETj2BSbqee0evGueE0jxyaibaiKI8=vI8tuQ8FMI8Gi=hEeeu0xXdbba9frFj0=OqFfea0dXdd9vqai=hGuQ8kuc9pgc9s8qqaq=dirpe0xb9q8qiLsFr0=vr0=vr0dc8meaabaqaciGacaGaaeqabaqadeqadaaakeaadaaeqbqaaiaadIhadaWgaaWcbaGaamyAaiaadQgaaeqaaOGaeyyzImRaaGymaaWcbaGaaiikaiaadMgacaGGSaGaamOAaiaacMcacqGHiiIZcaGGOaGaamiuamaaBaaameaacaWGTbaabeaaliaacYcacaWGqbWaaSbaaWqaaiaad6gaaeqaaSGaaiykaaqab0GaeyyeIuoaaaa@4653@,

for all interacting pairs of proteins {*P*_*m*_, *P*_*n*_}.

The variables (potential domain-domain interactions) and the constraints (interacting protein pairs to be explained) were coded into a sparse matrix, and the system was solved using an optimization toolbox in Matlab^® ^(The MathWorks Inc., Natick, MA, USA). Our LP had 177,233 variables and 26,032 constraints.

The noise in the protein-protein interaction data is modeled by randomizing the set of constraints. Namely, if we assume that the interactions are reliable with probability *r*, we include the corresponding constraint with probability *r*. We performed experiments setting the reliability at different rates. For each rate, the experiment was performed 1,000 times, with different numbers of constraints for each run, and the values obtained were averaged to generate the reported LP-score.

### Statistic measures

The pw-score for a given domain-domain interaction integrates two factors: the number of witnesses for the interaction and its 'promiscuity'. Let *w*(*i*,*j*) be the number of witnesses for a given domain pair (*i*,*j*) and let *r *be the assumed reliability of the network, that is, the probability that the interaction represented by an edge actually exists. The quantity (1 - *r*)^*w*(*i*,*j*) ^is the probability that all edges in the network that correspond to an interaction's witnesses are false positives. We compute the pw-score by taking the minimum between (1 - *r*)^*w*(*i*,*j*) ^and p value(*i*,*j*), an estimation of the influence of the frequency of appearance of the domain pair in its LP-score, computed as:

pw-score(*i*,*j*) = min(p value(*i*,*j*), (1 - *r*)^*w*(*i*,*j*)^)

The p values are computed in a separate randomization experiment. We create a set of 1,000 random networks assuming the same set of proteins with the same domain compositions, but selecting edges at random. The number of edges is kept the same but no other topological information is preserved. For each random network, we solve the corresponding LP formulation. For each domain pair, the p value is computed as a frequency of random network experiments that returned the LP-score at least equal to the LP-score obtained by the average of values in the 1,000 runs described above.

## Additional data files

The following additional data are available with the online version of this paper. Additional data file 1 is a list of the 3,000 top scoring domain pairs with a pw-score cutoff of 0.01 (network reliability 50%). Additional data file 2 is a list of the 3,000 top scoring domain pairs with a pw-score cutoff of 0.05 (network reliability 50%). Additional data file 3 is a list of the 3,610 domain pairs with a LP-score ≥ 0.4 and a pw-score ≤ 0.1 (network reliability 50%). Additional data file 4 is a list of the 3,944 domain pairs with a LP-score ≥ 0.4 and a pw-score ≤ 0.1 (network reliability 60%). Additional data file 5 is a list of the 783 gold standard domain pairs occurring in our dataset. Additional data file 6 is a list of the 1,780 interacting protein pairs used in the mediating domain pair prediction experiment. Additional data file 7 is a plot depicting the estimated sensitivity measures for the mediating domain pair prediction experiment.

## Supplementary Material

Additional data file 1 The 3,000 top scoring domain pairs with a pw-score cutoff of 0.01 (network reliability 50%). Click here for file

Additional data file 2 The 3,000 top scoring domain pairs with a pw-score cutoff of 0.05 (network reliability 50%). Click here for file

Additional data file 3 The 3,610 domain pairs with a LP-score ≥ 0.4 and a pw-score ≤ 0.1 (network reliability 50%). Click here for file

Additional data file 4 The 3,944 domain pairs with a LP-score ≥ 0.4 and a pw-score ≤ 0.1 (network reliability 60%). Click here for file

Additional data file 5 The 783 gold standard domain pairs occurring in our dataset. Click here for file

Additional data file 6 The 1,780 interacting protein pairs used in the mediating domain pair prediction experiment. Click here for file

Additional data file 7 The estimated sensitivity measures for the mediating domain pair prediction experiment. Click here for file

## Figures and Tables

**Table 1:** High-scoring pairs with a pw-score ≤ 0.01

Domain A	Domain B	Pfam A	Pfam B	LP-score	pw-score	GS	Diff	DPEA
IL8	7tm_1	PF00048	PF00001	1	0.0000			Yes
LSM	LSM	PF01423	PF01423	1	0.0000	Yes		Yes
Pkinase	Pkinase	PF00069	PF00069	1	0.0000	Yes		
Proteasome	Proteasome	PF00227	PF00227	1	0.0000	Yes		Yes
RRM_1	RRM_1	PF00076	PF00076	1	0.0000	Yes		Yes
zf-C2H2	zf-C2H2	PF00096	PF00096	1	0.0000	Yes		Yes
WD40	Cpn60_TCP1	PF00400	PF00118	1	0.0002			Yes
Pkinase	Cyclin_N	PF00069	PF00134	1	0.0004	Yes		Yes
zf-C3HC4	UQ_con	PF00097	PF00179	1	0.0004	Yes		Yes
RRM_1	LSM	PF00076	PF01423	1	0.0019			Yes
Chitin_bind_4	Chitin_bind_4	PF00379	PF00379	1	0.0039			Yes
TNFR_c6	TNF	PF00020	PF00229	1	0.0010	Yes	Yes	
PCI	PCI	PF01399	PF01399	0.999	0.0010		Yes	
Ras	Hrf1	PF00071	PF03878	0.999	0.0050			Yes
HATPase_c	HATPase_c	PF02518	PF02518	0.998	0.0050	Yes	Yes	
GTP_CDC	GTP_CDC	PF00735	PF00735	0.998	0.0010			Yes
Pfam-B_1	Nnf1	PB000001	PF03980	0.997	0.0070			Yes
Prefoldin	KE2	PF02996	PF01920	0.997	0.0100	Yes		Yes
C1-set	C1-set	PF07654	PF07654	0.996	0.0020	Yes	Yes	
Ferritin	Ferritin	PF00210	PF00210	0.996	0.0039	Yes		Yes
SH3_1	Pfam-B_18104	PF00018	PB018104	0.995	0.0010			Yes
Adap_comp_sub	Adaptin_N	PF00928	PF01602	0.994	0.0010	Yes		Yes
Globin	Globin	PF00042	PF00042	0.991	0.0040	Yes		Yes
BTB	BTB	PF00651	PF00651	0.99	0.0090	Yes	Yes	
WD40	Nrap	PF00400	PF03813	0.987	0.0090			Yes
EMP24_GP25L	EMP24_GP25L	PF01105	PF01105	0.986	0.0030	Yes		Yes
Pribosyltran	Pribosyltran	PF00156	PF00156	0.984	0.0030	Yes		Yes
Prenyltrans	PPTA	PF00432	PF01239	0.984	0.0020	Yes		Yes
Synaptobrevin	SNARE	PF00957	PF05739	0.982	0.0010	Yes	Yes	
V-SNARE	SNARE	PF05008	PF05739	0.976	0.0050	Yes	Yes	
bZIP	bZIP	PF00170	PF00170	0.976	0.0070	Yes		
Clat_adaptor_s	Adaptin_N	PF01217	PF01602	0.974	0.0030	Yes		Yes
Hexapep	Hexapep	PF00132	PF00132	0.973	0.0060	Yes		Yes
Autotransporter	Autotransporter	PF03797	PF03797	0.97	0.0000			Yes
CK_II_beta	CK_II_beta	PF01214	PF01214	0.968	0.0020	Yes		Yes
MCM	MCM	PF00493	PF00493	0.953	0.0000		Yes	
zf-U1	LSM	PF06220	PF01423	0.948	0.0080			Yes
Ribonuc_red_sm	Ribonuc_red_sm	PF00268	PF00268	0.944	0.0010	Yes		Yes
SNARE	SNARE	PF05739	PF05739	0.943	0.0000	Yes	Yes	
CBFD_NFYB_HMF	CBFD_NFYB_HMF	PF00808	PF00808	0.942	0.0040	Yes		Yes
SNARE	Sec1	PF05739	PF00995	0.941	0.0020	Yes	Yes	
ubiquitin	UBA	PF00240	PF00627	0.94	0.0090		Yes	
IF-2B	IF-2B	PF01008	PF01008	0.94	0.0060	Yes		Yes
KH_1	KH_1	PF00013	PF00013	0.94	0.0090	Yes	Yes	
Chorion_3	CBM_14	PF05387	PF01607	0.939	0.0050			Yes
SH3_1	Pfam-B_62907	PF00018	PB062907	0.936	0.0010		Yes	
Clat_adaptor_s	Adap_comp_sub	PF01217	PF00928	0.935	0.0030	Yes		Yes
Bac_DNA_binding	Bac_DNA_binding	PF00216	PF00216	0.933	0.0010	Yes		Yes
Cyclin_N	CKS	PF00134	PF01111	0.933	0.0090		Yes	

Columns GS, Diff, and DPEa indicate, respectively, if the pair is in the gold standard set, if it is difficult (does not have a witness), and if it was predicted among the high-confidence pairs by the DPEA method. Among these 50 predictions, only 17 are not in the gold standard set. Out of the 14 pairs that are in the difficult category, nine are confirmed by crystal structures, three have been inferred to interact in literature [28-30], and one is between a PFAM-A and a PFAM-B domain (thus no literature evidence is expected). The last one, involving cyclin and cyclin-dependent kinase regulatory subunit (CKS), has been investigated by Aloy and Russell [31], and may represent a wrong prediction introduced by an error in the high-throughput data.

**Table 2:** High-scoring partners of Ras and SNARE domains (pw-score ≤ 0.05)

Domain A	Domain B	Pfam A	Pfam B	LP-score	pw-score	GS	Diff	DPEA
Ras	Yip1	PF00071	PF04893	1	0.035			Yes
Ras	GDI	PF00071	PF00996	1	0.037	Yes		Yes
Ras	Hrf1	PF00071	PF03878	0.999	0.005			Yes
Ras	Rho_GDI	PF00071	PF02115	0.871	0.002	Yes		Yes
Ras	TBC	PF00071	PF00566	0.773	0.022		Yes	
Ras	Peptidase_M18	PF00071	PF02127	0.765	0.014			Yes
Ras	Mss4	PF00071	PF04421	0.762	0.019		Yes	
Ras	PBD	PF00071	PF00786	0.711	0.013		Yes	
Ras	Y_phosphatase2	PF00071	PF03162	0.677	0.027			
Ras	IF4E	PF00071	PF01652	0.675	0.039			Yes
Ras	Porin_3	PF00071	PF01459	0.673	0.047			
Ras	NAC	PF00071	PF01849	0.61	0.019			
Ras	RasGAP	PF00071	PF00616	0.545	0.002	Yes	Yes	
Ras	SNARE	PF00071	PF05739	0.545	0.042		Yes	
Ras	PMM	PF00071	PF03332	0.528	0.007			Yes
Ras	Hexapep	PF00071	PF00132	0.519	0.046			
Ras	DHO_dh	PF00071	PF01180	0.516	0.01			Yes
Ras	Arginase	PF00071	PF00491	0.516	0.011			Yes
Ras	Thi4	PF00071	PF01946	0.514	0.006			Yes
Ras	Pept_C1-like	PF00071	PF03051	0.514	0.01			Yes
Ras	AA_kinase	PF00071	PF00696	0.513	0.008		Yes	
Ras	Glyco_hydro_47	PF00071	PF01532	0.513	0.025			
Ras	Pfam-B_5516	PF00071	PB005516	0.512	0.005			
Ras	UDPGT	PF00071	PF00201	0.512	0.045			Yes
Ras	Pfam-B_17923	PF00071	PB017923	0.511	0.009			Yes
Ras	Aminotran_3	PF00071	PF00202	0.511	0.041			
Ras	Pfam-B_90255	PF00071	PB090255	0.51	0.006			Yes
Ras	F_actin_cap_B	PF00071	PF01115	0.509	0.026			Yes
Ras	dUTPase	PF00071	PF00692	0.508	0.032			Yes
Ras	Cpn10	PF00071	PF00166	0.507	0.021			Yes
Ras	NIF3	PF00071	PF01784	0.505	0.02			Yes
Ras	NDK	PF00071	PF00334	0.505	0.025			Yes
Ras	ALAD	PF00071	PF00490	0.503	0.003			Yes
Ras	Pfam-B_52661	PF00071	PB052661	0.501	0.01			Yes
Ras	Pfam-B_99124	PF00071	PB099124	0.501	0.012			Yes
SNARE	Synaptobrevin	PF05739	PF00957	0.982	0.001	Yes	Yes	
SNARE	V-SNARE	PF05739	PF05008	0.976	0.005	Yes	Yes	
SNARE	SNARE	PF05739	PF05739	0.943	0	Yes	Yes	
SNARE	Sec1	PF05739	PF00995	0.941	0.002	Yes	Yes	
SNARE	Adaptin_N	PF05739	PF01602	0.858	0.003		Yes	
SNARE	MAP1_LC3	PF05739	PF02991	0.596	0.001		Yes	
SNARE	Ras	PF05739	PF00071	0.545	0.042		Yes	
SNARE	Prenyltrans	PF05739	PF00432	0.518	0.005			Yes

Prediction of Ras and SNARE interactions with a LP-score ≥ 0.5 and a pw-score ≤ 0.05. Out of 35 putative Ras interactions, six are difficult, three (among them one difficult) are documented by a crystal structure. More than 70% of easy predictions belong to the high-confidence DPEA predictions. The interaction between Ras and Mss4 is known from literature, with the caveat discussed in the text. All but one of our predictions of SNARE interactions are in the difficult category. Of the predictions above a LP-score of 0.6, all but one are documented with crystal structure. Columns GS, Diff, and DPEa indicate, respectively, if the pair is in the gold standard set, if it is difficult (does not have a witness), and if it was predicted among the high-confidence pairs by the DPEA method.
