# Supplementary figures and images for: Predicting domain-domain interactions using a parsimony approach
Source: Genome Biol. 2006 Nov 9;7(11):R104. doi: 10.1186/gb-2006-7-11-r104 (PMC1794579; doi:10.1186/gb-2006-7-11-r104)

Comparison of Sensitivity in Mediating Domain Pair Prediction Experiment

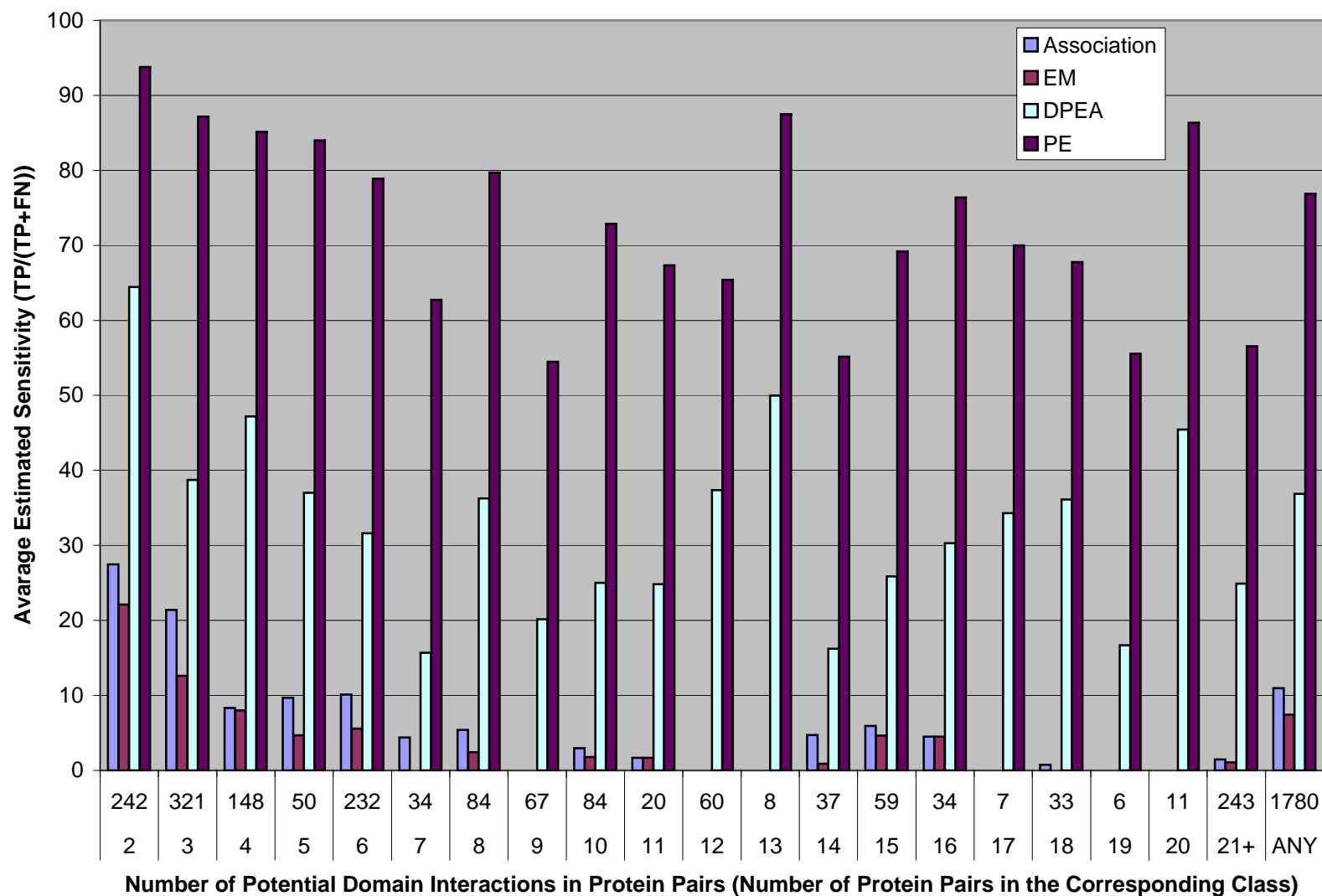

Supplement: Additional data file 7 — The estimated sensitivity measures for the mediating domain pair prediction experiment. [file gb-2006-7-11-r104-S7.pdf]
